# A Case of Unusual Guidewire Tip Entrapment During Side Branch Recrossing: Successful Retrieval With the Proximal Optimization Technique

**DOI:** 10.1016/j.jscai.2026.105310

**Published:** 2026-04-09

**Authors:** Bayushi Eka Putra, Takuro Sugie, Yoshifumi Kashima, Shoichi Kuramitsu

**Affiliations:** a Department of Cardiovascular Medicine, Sapporo Cardiovascular Clinic, Sapporo Heart Center, Sapporo, Japan; b Department of Cardiology, Berkah General Hospital Pandeglang, Kabupaten Pandeglang, Banten, Indonesia; c Department of Cardiology, Oita Cardiovascular Hospital, Oita, Japan

**Keywords:** case report, complications, guidewire entrapment, percutaneous coronary intervention

## Abstract

Coronary guidewire entrapment is a rare but serious complication that can occur during percutaneous coronary intervention. We report an unusual case of guidewire tip entrapment that occurred during an attempt to recross into a side branch after stent implantation. In this case, only the coil tip remained trapped after repeated microcatheter-assisted retrieval attempts, and it was unexpectedly dislodged into the aorta during reproximal optimal technique procedures. Based on this experience, we propose the proximal optimal technique as a potential bailout strategy when conventional approaches fail in cases of guidewire tip entrapment.

## Introduction

Coronary guidewire entrapment is a rare but serious complication that can occur during percutaneous coronary intervention (PCI).^1^^,^^2^ The overall incidence of this complication is reported to be approximately 0.1% to 0.2%, although the frequency increases in complex PCI cases.^1^^,^^2^ Guidewire entrapment typically occurs following excessive manipulation within calcified or tortuous lesions, jailing of the guidewire during bifurcation stenting, or during complex PCI for chronic total occlusion lesions.^1^^,^^2^ Here, we report a rare case of guidewire tip entrapment that occurred during side branch recrossing and was unexpectedly dislodged into the aorta during re-proximal optimal technique (POT) procedures. We propose POT as a potential bailout strategy for guidewire tip entrapment when conventional approaches are unsuccessful.

## Case presentation

**Figure 1:**
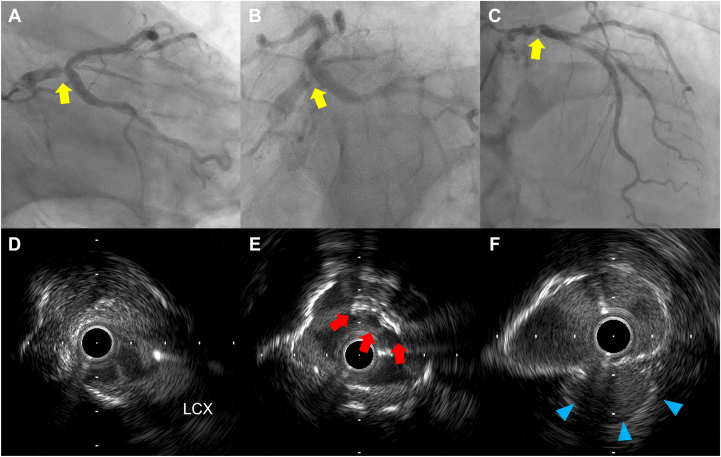
**Angiographic and intravascular ultrasound findings.** (**A-C**) Preangiographic findings. (**D-F**) Preintravascular ultrasound (IVUS) findings from the distal (**D**) to the proximal sites (**F**). IVUS demonstrates a calcified nodule (E; red arrows) and plaque rupture (F; blue arrows). IVUS, intravascular ultrasound; LCX, left circumflex coronary artery.

**Figure 2:**
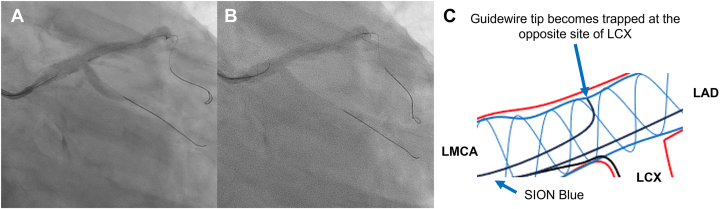
**Occurrence of guidewire tip entrapment.** (**A**) Angiographic image after the proximal optimization technique. (**B**, **C**) Guidewire tip entrapment occurred during an attempt to recross into a left circumflex coronary artery (LCX). LAD, left anterior descending coronary artery; LCX, left circumflex coronary artery; LMCA, left main coronary artery.

**Figure 3:**
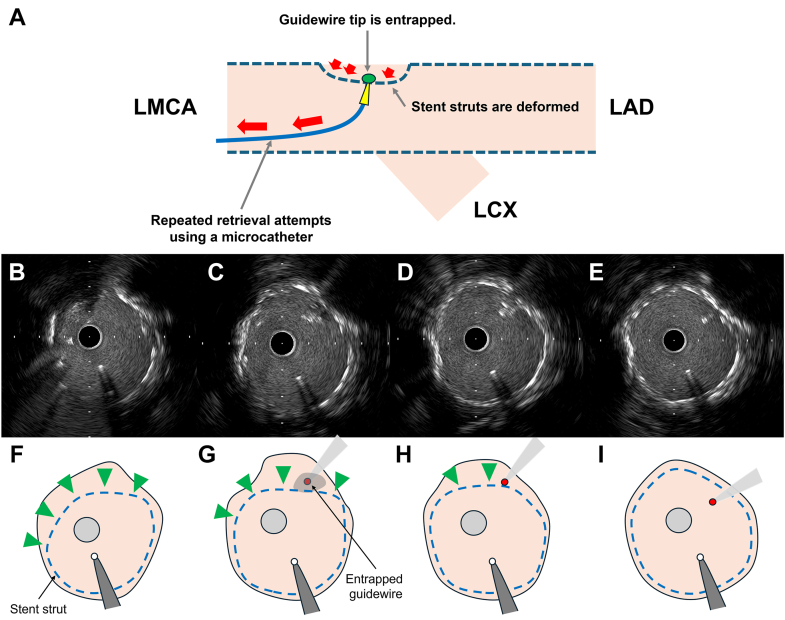
**Intravascular ultrasound findings after failed microcatheter-assisted retrieval of an entrapped guidewire tip.** (**A**) Underlying mechanism of stent deformation. (**B-E**) Serial IVUS images from the distal (**B**) to the proximal (**E**) LMCA. Panels F, G, H, and I correspond to panels **B**, **C**, **D**, and **E**, respectively. (**F**-**H**) Stent deformation by repeated microcatheter-assisted retrieval and stent malappositions (green arrowheads). (**G-I**) The entrapped guidewire tip (small red circle) was observed from the area around the malapposed struts with low-echogenic tissues (**F**) to the proximal segment of the LMCA. IVUS, intravascular ultrasound; LAD, left anterior descending coronary artery; LCX, left circumflex coronary artery; LMCA, left main coronary artery.

An 84-year-old man with unstable angina pectoris underwent coronary angiography, revealing a significant stenosis at the distal left main coronary artery (Figure 1A-C). Intravascular ultrasound (IVUS) confirmed plaque rupture with calcified nodules (Figure 1D-F; Supplemental Video 1). Intravascular lithotripsy was performed with a 4.0 × 12 mm balloon, achieving full balloon expansion after 80 impulses. A 4.0 × 33 mm drug-eluting stent (DES) (XIENCE Skypoint, Abbott) was implanted, followed by a 5.0 × 15 mm noncompliant balloon after dilatation, yielding good results (Figure 2A). Afterward, we attempted to recross a guidewire (SION Blue, Asashi Intecc) into the left circumflex artery for a kissing balloon inflation. Despite gentle manipulation, the guidewire tip unintentionally became trapped at the opposite side of the left circumflex coronary artery (Figure 2B, C). Advancing a microcatheter (Corsair Pro, Asashi Intecc) over the trapped guidewire was initially attempted but failed to retrieve it (Supplemental Video 2). Repeated retrieval attempts using the microcatheter led to fracture of the spring coil, leaving only the coil tip lodged in place (Supplemental Video 3). As IVUS revealed DES deformation caused by several retrieval attempts (Figure 3; Supplemental Video 4), re-POT was performed using a 5.5 × 12 mm noncompliant balloon at 22 atm. Unexpectedly, the trapped wire tip—resistant to microcatheter retrieval—became dislodged proximally into the aorta during re-POT procedures (Supplemental Video 5). The dislodged guidewire tip was found in the distal aorta and successfully retrieved with a snare device via the femoral artery (Supplemental Video 6).

## Discussion

**Figure 4:**
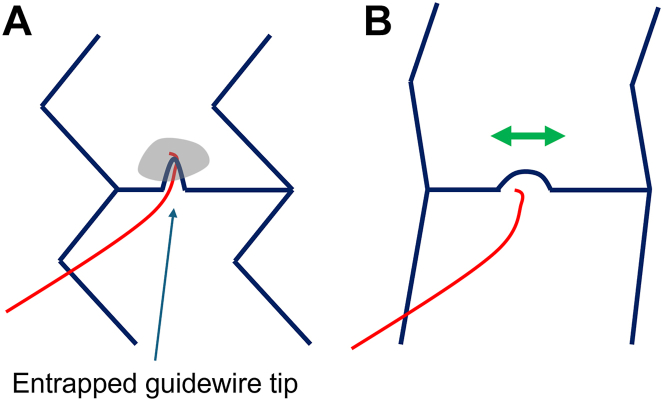
**Possible mechanism of releasing the dislodged guidewire tip after the reproximal optimization technique.** (**A**) The guidewire tip is entrapped at the longitudinal connector. (**B**) The reproximal optimization technique stretches the longitudinal connector and thereby releases the entrapped guidewire tip.

The occurrence of guidewire entrapment is typically associated with aggressive manipulation within complex lesions or with jailing of the guidewire during bifurcation stenting.^1^^,^^2^ In this case, a workhorse wire was used and manipulated gently. Despite this, the standard bailout approach—microcatheter-assisted retrieval—failed to retrieve the entrapped tip. Consequently, only the coil tip remained trapped after an attempted retrieval using a microcatheter and was unexpectedly dislodged into the aorta during re-POT procedures. These observations suggest that the guidewire tip may have been entrapped within not only the coronary plaque but also within the stent struts. The XIENCE Skypoint platform features a peak-to-valley 3-link design. Although IVUS could not provide a detailed visualization of the stent struts, the guidewire tip might have been entrapped at a longitudinal connector (Figure 4A). As the re-POT was performed with a 5.5-mm balloon inflated to 22 atm, the 4.0-mm XIENCE Skypoint could have reached its overexpansion capacity (5.75 mm). This re-POT-induced stent expansion could have stretched the longitudinal connector, thereby releasing the entrapped guidewire tip (Figure 4B).

Bailout techniques for guidewire entrapment include the use of a second wire, balloon-assisted retrieval, guide catheter extension, microcatheter, or even a snare.^1^^,^^2^ However, when standard retrieval techniques fail, the management of the entrapped guidewire becomes particularly challenging. The observations from the present case suggest that POT may serve as an alternative approach when the guidewire tip is entrapped within stent struts. In addition, employing a ping-pong technique—one guiding catheter securing the entrapped guidewire tip while another is used for POT—may help minimize the risk of a dislodged guidewire. However, it should be noted that POT has the potential to worsen the situation when the guidewire tip is entrapped within coronary plaque rather than the stent struts. Therefore, operators should carefully consider the most appropriate management strategy based on the underlying mechanism of the guidewire entrapment.

## Conclusion

Coronary guidewire entrapment is a rare but serious complication that can occur during complex PCI. POT may serve as an alternative strategy for managing guidewire entrapment when standard bailout approaches fail.

## Declaration of competing interest

The authors declared no potential conflicts of interest with respect to the research, authorship, and/or publication of this article.

## Footnotes

To access the supplementary material accompanying this article, visit the online version of the *Journal of the Society for Cardiovascular Angiography & Interventions* at 10.1016/j.jscai.2026.105310.
